# Increased Level of Phosphorylated ShcA Measured by Chemiluminescence-Linked Immunoassay Is a Predictor of Good Prognosis in Primary Breast Cancer Expressing Low Levels of Estrogen Receptor

**DOI:** 10.3390/cancers2010153

**Published:** 2010-03-12

**Authors:** Jonas Cicenas, Willy Küng, Urs Eppenberger, Serenella Eppenberger-Castori

**Affiliations:** 1 Stiftung Tumorbank Basel, Basel, Switzerland; E-Mail: u.eppenberger@tumorbank.org (U.E.); 2 Molecular Tumor Biology, Department of Research, Universitätsspital, Basel, Switzerland; E-Mail: willy.kueng@unibas.ch (W.K.); 3 Molecular Pathology, Institut für Pathologie, Universitätsspital, Basel, Switzerland; E-Mail: sere.eppenberger@unibas.ch (S.E.)

**Keywords:** ShcA, immunoassay, kinase, biomarker, estrogen receptor, phosphorylation, breast cancer

## Abstract

The SH2 domain-containing adaptor protein ShcA is a proto-oncogene involved in growth factor receptor signaling. The role of phosphorylated ShcA is to link receptor tyrosine kinases with the SH2-containing adaptor protein Grb2, thus facilitating signal transduction from receptor tyrosine kinases to Ras, leading to MAPK activation. The present study was designed to investigate the prognostic significance of phosphorylated ShcA in primary breast cancer and its association in the interactions between the ER and ErbB2 pathways. Using a two-site chemiluminescence-linked immunosorbent assay, we detected the quantitative expression levels of total tyrosine- and threonine-phosphorylated ShcA in cytosol fractions obtained from fresh frozen tissue samples of 153 selected primary breast cancer patients. ShcA phosphorylation was not associated with nodal status, estrogen receptor (ER) status or grading. High levels of both tyrosine (pYShcA) and serine (pSShcA) phosphorylated ShcA correlated with good prognosis (p < 0.01), with respect to both disease-free (DFS) and overall survival (OS). In addition, pShcA levels were found to correlate with threonine-phosphorylated ErbB2 and inversely with phosphorylated Akt (pAkt), as well as ErbB2 and ER expression levels. Our findings demonstrate that ShcA activation in primary breast cancer patients correlates with low levels of ER, and is associated with good prognosis.

## 1. Introduction

Molecular analysis of human tissue specimens is performed for diagnostic and prognostic purposes and provides valuable data about the expression of individual proteins or genes. Such an approach, however, remains restricted in its ability to reveal the function of such proteins. Protein function is regulated not only by transcription and translation, but also by posttranslational modifications such as phosphorylation, glycosylation, and acetylation. Phosphorylation is involved in most cell signal transduction pathways, and is thus, essential for both normal physiology and disease. Therefore, the number of proteins in the phosphorylated state may be more biologically significant than the total number of proteins present. We have previously shown that protein phosphorylation can improve the prognostic value of Akt and ErbB2 proteins [1,2].

The adaptor protein Shc is a SH2 domain-containing proto-oncogene implicated in growth factor signaling. In addition to the ubiquitously expressed ShcA, two other isoforms exist - ShcB and ShcC, which are expressed in neuronal cells. Biochemical and genetic data revealed a general role of ShcA proteins in the transduction of signals from receptor tyrosine kinases to Ras, leading to MAPK activation [3,4].

ShcA can be expressed as three splice forms of approximately 46, 52 and 66 kDa. p46 and p52 ShcA isoforms share an amino-terminal SH2 domain, followed by a CH domain, and a carboxy-terminal phosphotyrosine-binding domain (PTB). Within the CH domain of ShcA, three tyrosine-phosphorylation sites have been identified. The role of phosphorylated ShcA proteins is to link receptor tyrosine kinases with the SH2-containing Grb2 adaptor protein [5,6,7]. The PTB and/or SH2 domains of ShcA bind to tyrosine-phosphorylated receptors, while the SH2 of Grb2 binds the tyrosine-phosphorylated ShcA. Grb2 subsequently becomes constitutively bound to Sos, a Ras guanine nucleotide exchange factor. Recruitment of the Grb2/Sos complex by ShcA results in the membrane relocalization of Sos, following Ras activation. Nevertheless, whether ShcA is essential in Ras activation is disputed. On one hand, the Grb2/Sos complex can be recruited directly to activated receptors with subsequent Ras activation. In addition, in cells derived from ShcA knockout mouse embryos, Ras activation seems to be undisturbed [8]. It has been suggested that ShcA might serve as an enhancer of receptor tyrosine signaling at low concentrations of growth factors. Still, the defined role of ShcA in the activation of Ras remains unclear.

p66 ShcA is a third isoform expressed through alternative splicing [9]. It contains the entire p52 ShcA sequence and an additional domain, similar to a CH domain, termed CH2. In spite of its tyrosine-phosphorylation by active receptor tyrosine kinases, p66 ShcA is not implicated in - and seems even to inhibit - Ras activation [10]. The CH2 domain contains a serine phosphorylation site that is involved in oxidative stress signaling [11].

In the present study, we report serine and tyrosine phosphorylated ShcA (pSShcA and pYShcA, respectively) by means of a novel two-site chemiluminescence-linked immunoassay (CLISA) in fresh frozen primary tissue samples from 153 selected primary breast cancer patients. The aim of this study was to investigate the prognostic value of phosphorylated ShcA, as well as its association in the interactions between the ER and ErbB2 pathways.

## 2. Results and Discussion

### 2.1. Distribution of Phosphorylated ShcA Levels and Their Correlation with Tumor Characteristics

**Figure 1:**
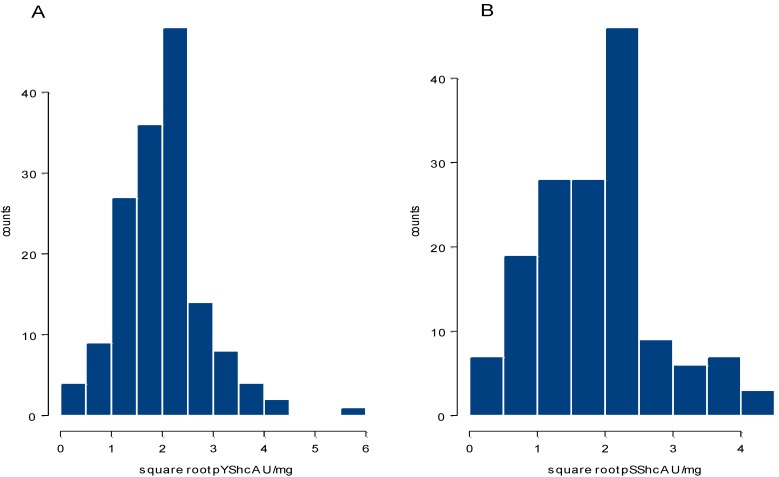
Histograms showing distribution of square root values of (**A**). pYShcA and (**B**). pSShcA expression levels in 153 primary breast cancer samples by CLISA.

CLISA quantified pShcA levels in the 153 primary breast cancer samples studied have a left-tailed distribution ranging from 0–32.33 U/mg with a median of 4.05 U/mg (mean 4.63 U/mg) for pYShcA and from 0–19.49 U/mg with a median of 3.76 U/mg (mean 4.27 U/mg) for pSShcA. After square root transformation, values become almost normally distributed (Figure 1). In this set of primary breast cancer samples, we did not find any significant difference in pShcA levels with respect to nodal status, ER-status or grading. pYShcA had a slightly inverse correlation with tumor size (r_s_ = −0.19, p = 0.02), while no correlation was found between pSShcA and tumor size. Surprisingly, pShcA inversely correlated with ER continuous values in this subset of patients selected according to their low ER expression levels (Table 1).

**Table 1:** Spearman rank correlations of quantitative pShcA and of other biomarkers expression levels.

	ErbB2	pTErbB2	pAkt	ER	pYShcA	pSShcA
**pYShcA**	r_s_ = -0.17	r_s_ = 0.27	r_s_ = -0.27	r_s_ = -0.16	r_s_ = 1	r_s_ = 0.88
	(p = 0.03)	(p = 0.01)	(p = 0.01)	(p = 0.05)		(p < 0.001)
**pSShcA**	n.s.	r_s_ = 0.21	r_s_ = -0.28	r_s_ = -0.16	r_s_ = 0.88	r_s_ = 1
		(p = 0.01)	(p < 0.001)	(p = 0.05)	(p < 0.001)	

### 2.2. Correlation of Phosphorylated ShcA Levels and Levels of Other Tumor Markers

**Figure 2:**
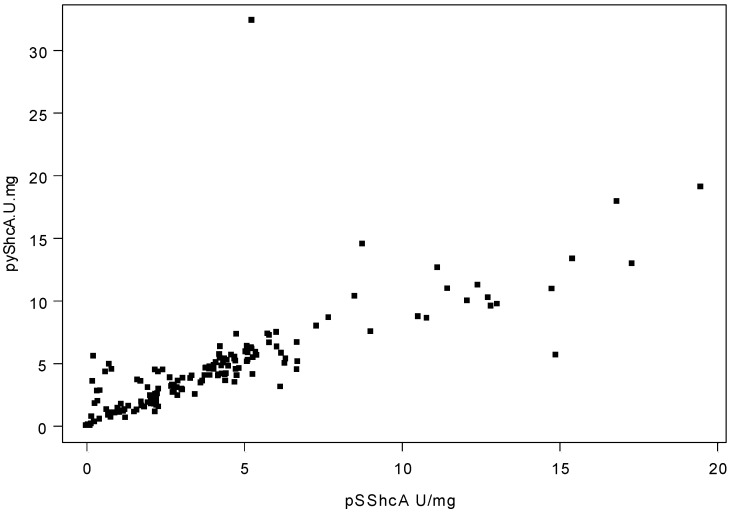
Scatter plot of pYShcA versus pSShcA expression levels (r_s_ = 0.88, p < 0.001).

We compared the quantitative pShcA protein levels to the quantitative protein expression or phosphorylation levels of other markers assessed in our laboratory. Using Spearman rank correlation, pShcA levels were found to correlate with threonine-phosphorylated ErbB2 (pTErbB2) levels and inversely with phosphorylated Akt (pAkt) levels as well as ErbB2 and ER expression levels (Table 1). There also was a very strong correlation between pYShcA and pSShcA levels (r_s_ = 0.88, p < 0.001) (Figure 2).

### 2.3. Prognostic Significance of Tyrosine Phosphorylated ShcA

**Figure 3:**
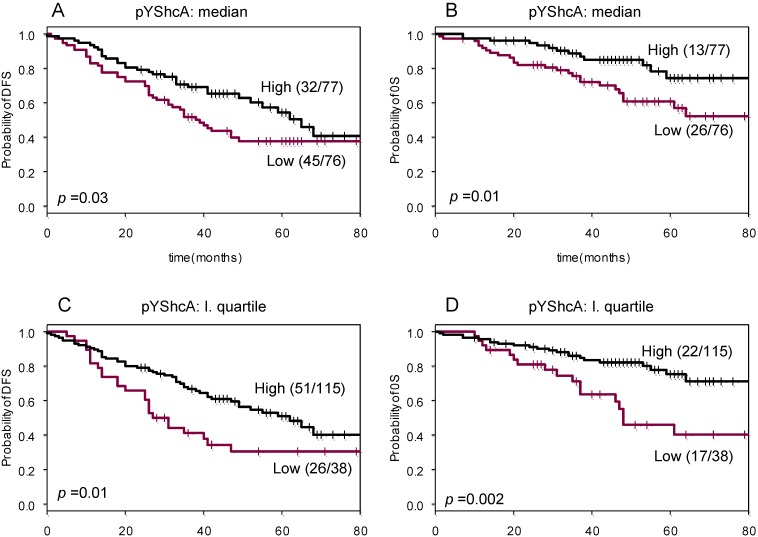
Kaplan–Meier survival curves for pYShcA. Patients were dichotomized according to the median (**A**, **B**) or first quartile expression value of pYShcA (**C**, **D**). Disease-free survival (DFS) (**A**, **C**) and overall survival (OS) in (**B**, **D**).

The prognostic value of pYShcA was investigated with respect to DFS and OS. (Figure 3). Univariate Cox regression revealed a weak and significant (*p* < 0.05; likelihood ratio test) correlation between pYShcA levels and DFS and OS. Median value of pYShcA was used as a cut-off value for pYShcA status. Subsequently, Kaplan–Meier survival analysis was performed for pYShcA-negative versus pYShcA-positive tumors. Forty-five out of 76 (59%) of patients with pYShcA-negative tumors developed disease recurrence, whereas this was the case for only 32 out of 77 (42%) in the pYShcA-positive group (*p* = 0.03). In the OS analysis, 26 out of 76 (34%) patients with pYShcA-negative tumors died as compared to 13 out of 77 (17%) in pYShcA-positive tumors (*p* = 0.01).

This effect was more pronounced when pYShcA levels exceeding the first quartile value were used as a cut-off value. Twenty-six out of 38 (68%) patients with pYShcA-negative tumors developed disease recurrence, while this was the case for only 51 out of 115 (44%) in the pYShcA-positive group (*p* = 0.007). In the OS analysis, 17 out of 38 (45%) patients with pYShcA-negative tumors died as compared to 22 out of 115 (19%) in pYShcA-positive tumors (*p* = 0.002).

### 2.4. Prognostic Significance of Serine Phosphorylated ShcA

**Figure 4:**
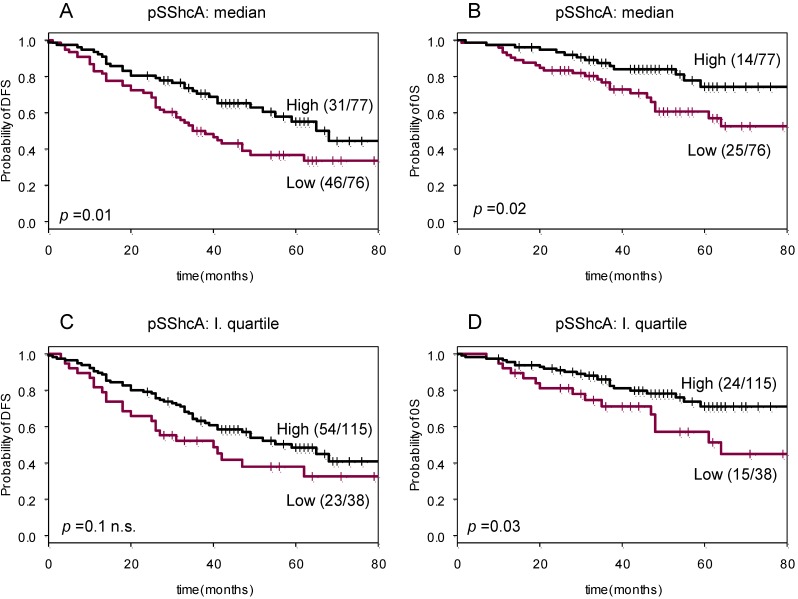
Kaplan–Meier survival curves for pSShcA. Patients were dichotomized according to the median (**A**, **B**) or first quartile expression value of pSShcA (**C**, **D**). Disease-free survival (DFS) (**A**, **C**) and overall survival (OS) in (**B**, **D**).

Univariate Cox regression revealed also a weak significant (*p* < 0.05; likelihood ratio test) correlation between pSShcA levels and survival. Median value of pSShcA was used as a cut-off value for pSShcA status. Subsequently, Kaplan–Meier survival analysis was performed for pYShcA-negative *versus* pYShcA-positive tumors (Figure 4 A, B). Forty-six out of 76 (61%) patients with pSShcA-negative tumors developed disease recurrence, whereas this was only the case for 31 out of 77 (40%) in the pSShcA-positive group (*p* = 0.01). In the OS analysis, 25 out of 76 (33%) patients with pSShcA-negative tumors died as compared to 14 out of 77 (18%) in pSShcA-positive tumors (*p* = 0.02).

Using the first quartile value of pSShcA levels as threshold, the association with survival observed was weaker (Figure 4 C, D). Twenty-three out of 38 (61%) patients with pSShcA-negative tumors developed disease recurrence and 54 out of 115 (47%) in the pSShcA-positive group (n.s.). In the OS analysis, significance was observed since 15 out of 38 (40%) patients with pSShcA-negative tumors died as compared to 24 out of 125 (21%) in pYShcA-positive tumors (*p* = 0.03).

### 2.5. Discussion

This is the first study to measure quantitative levels of phosphorylated ShcA applying a newly developed immunoassay, and investigating the relationship to protein expression levels of the established prognostic markers and survival.

Phosphorylation, which actually reflects the functionality of various proteins, has a potential to be of better prognostic value than expression levels of those proteins. Subsequently, the analysis of phosphorylation of important signaling molecules is becoming more and more prominent. Phosphorylation of proteins, such as Akt [1,12,13], ErbB2 [2,13,14], p21Cip1 [15], p27KIP1 [16] and others, have already been shown to have a prognostic value in breast cancer.

Our study is the first report on pShcA assessed quantitatively using a phospho-specific antibody in breast cancer cytosols of cryopreserved tumor samples. We also understand the limits of our assays, since the conditions of our assay do not entirely rule out co-immunoprecipitation, therefore we cannot exclude that the measured phosphoproteins could in part represent ShcA binding proteins. Moreover, it should be noted that anti-phosphoserine antibodies do not recognize all serine phosphorylation sites, and different anti-phosphoserine antibodies may possibly recognize different epitopes on the same protein. Therefore, the results are related to specific epitopes recognized by the selected antibodies. However, the CLISA assay used in this study was reliable and repeated measurements revealed a highly reproducibility. Measurements were performed on homogenized samples, which were checked by the pathologist in charge and confirmed to contain at least 60% tumor cells. In addition, samples were previously analyzed for ER, PgR and ErbB2 using both EIA assays, immunohistochemistry and/or fluorescence *in situ* hybridization, and a good correlation between the assays was observed [17], implying that sample homogenization does not play a role in the final results.

pYShcA and pSSchA correlated very strongly to each other. Univariate Cox-regression revealed a statistical significant correlation between pShcA and patient survival. Kaplan-Meyer curves were plotted using the median and the first quartile values of both test results. In general, patients with tumors expressing higher levels of pYShcA and/or pSSchA, though lower levels of ER had significantly better prognosis.

It is noteworthy that we selected the samples a priori according to ER and ErbB-2 protein expression levels and explicitly enriched the population with ER-negative and ErbB-2 positive samples (64% ER negative and 53% ErbB-2 positive instead of ~30% and 15%, respectively, in a randomized population). Moreover, our ER-positive population had ER protein expression levels ranging from 10-393 fmol/mg total protein. This corresponds to the lower third of ER expression levels among ER- positive patients when compared to the distribution of ER protein expression levels observed in a large randomized study population, where ER expression levels among ER positive patients range from 1-1200 fmol/mg. Following our findings confirm that elevated pShcA is providing additional prognostic information for patients with tumors expressing lower ER and higher ErbB-2 levels.

The original finding of this study is that pShcA expression seems to have a favorable effect on patient survival. This is in disagreement with a number of *in vitro* data, showing that the RAS-MAPK pathway is involved in cellular proliferation and motility. Besides, there are several studies dealing with ERK1/2 phosphorylation as a good prognosis predictor in different types of human cancers, particularly in breast. One study in breast cancer demonstrated by immunohistochemistry that high pERK expression correlated with early stages, negative nodal status and long recurrence-free survival, and that high ERK phosphorylation levels was an independent indicator of long relapse-free survival and overall survival in multivariate analysis [18]. Another study in breast cancer suggests that pERK expression is associated with small tumors with better survival [19]. Yet another breast cancer study showed that positive pERK1/2 indicated better DFS in patients treated with tamoxifen [20]. Based on these findings and others, it is consistent to assume that pShcA, being upstream of ERK1/2, can be recognized as a good prognosis factor, most probably acting in the same pathway.

In addition, the correlation of pShcA to the threonine phosphorylated ErbB2 is also very interesting. We have some indications that serine and threonine phosphorylated ErbB2 have potential prognostic values (data not shown).

## 3. Experimental

### 3.1. Tumor and Patient Characteristics

A collective of 153 cryopreserved (−80 °C) and well characterized primary breast tumors was selected from our tumor bank according to their ErbB2 and ER protein expression levels routinely assessed at the time of surgery. In order to better understand the role of pSchcA in the amplification of ErbB2 or down-modulation of ER, we selected tumors with high ErbB2 or low ER expression levels. Eighty-one (53%) of tumors overexpressed ErbB2, the remaining 72 (47%) had intermediate (50–260 ng/mg) levels. Ninety-eight tumors (64%) were ER negative, the remaining 55 (36%) had low (20–390 fmol/mg) ER levels. All patients underwent primary surgery before January 1996. Seventy-seven (50%) patients experienced a disease recurrence within the median follow-up time of 47 months (range 24–87 months). Fifty-eight patients (38%) were nodal negative and 75 (49%) nodal positive. None of the patients received neoadjuvant therapy. Patients and tumor characteristics are summarized in Table 2.

**Table 2:** Clinicopathological characteristics of the patients.

Feature		Number of patients (%)
Patients enrolled		153
Age	<40	10 (6.5)
	40–60	88 (57.5)
	>60	55 (36)
Histology type		
	Ductal	104 (68)
	Lobular	9 (6)
	Other	40 (26)
Tumor size		
	T1	39 (25)
	T2	87 (57)
	T3-4	24 (16)
	Unknown	3 (2)
Lymph-node status		
	Node-negative	58 (38)
	Node-positive	75 (49)
	Unknown	20 (13)
Histopathological grade		
	I + II	49 (32)
	III	57 (37)
	Not analyzed	47 (31)
Estrogen receptor (ER)		
	Positive (>20 fmol/mg)	55 (36)
	Negative (≤20 fmol/mg)	98 (64)
Progesterone receptor (PgR)		
	Positive (>20 fmol/mg)	40 (26)
	Negative (≤20 fmol/mg)	113 (73)
ErbB2		
	Positive (>260 ng/mg)	81 (53)
	Negative (≤260 ng/mg)	72 (47)

### 3.2. Cell Lines and Tissue Preparation

SKBr3 breast cancer cells were cultured in Improved Minimal Essential medium with zinc option (IMEM-ZO) supplemented with 5% fetal bovine serum and L-glutamine at 37 °C in a 5% CO_2_ incubator. For the phospho-standard preparation, sub-confluent SKBr3 cells were serum-starved for 48 h in serum free medium, treated with NaF and Na_3_VO_4_ for 1 h, then with 10% FBS for 10 min. Cells were lysed in EB lysis buffer (0.5 M NaCl, 10 mM EDTA, pH8, 1% Triton X100, 20 mM Tris-HCl, pH7.0, 20 mM NaF, 20 mM glycerophosphate, 2 mM Na_3_VO_4_, proteinase inhibitor cocktail, Roche) for 5 min on ice, centrifuged at 20,000 g for 5 min and the supernatant stored at −80 °C. 

### 3.3. Measurement of ER, PgR and ErbB-2 Protein Levels in Tumor Extracts by EIA

Tissue homogenates were prepared in accordance with standard procedures for tumor marker EIA measurement as previously described [17,1,2]. In brief, the frozen tissues were pulverized in liquid nitrogen using a Micro-Dismembrator U (B. Braun Melsungen AG, Melsungen, Germany). The powder was homogenized with a tissue homogenizer (Ultra-Turrax; Janke & Kunkel, IKA-Werke, Staufen, Germany) for 20 seconds in three volumes of ice-cold extraction buffer. The homogenate was briefly centrifuged, and the supernatant was centrifuged again in an ultracentrifuge (Beckman Instruments, Fullerton, CA) at 100,000 g. The resulting supernatants (cytosols) were used for measurement of the hormone receptors (ER, PgR), and the membrane fractions for EIA measurement of membrane-associated ErbB-2. ER and PgR concentrations were measured from tumor cytosolic extracts by commercial quantitative ER and PgR EIA kits (Abbott Laboratories, Abbott Park, IL) using a Quantum II photometer. Quality control of ER and PgR measurement were carried out in collaboration with the Receptor Biomarker Group (RBG) of the European Organization for Research and Treatment of Cancer (EORTC) [21]. ErbB-2 receptor levels were determined on the particulate membrane fractions of tumor extracts using a commercial monoclonal antibody EIA kit at the time of surgery as described [17]. Tumors with ERBB2 protein levels >260 ng/mg total protein were considered as positive, which corresponds to the previously published [17] cut-off value of 500 U/mg total protein and correlates with the immunohistochemistry (IHC) DAKO 3 + 8 as well as ERBB2 amplification detected by fluorescence in situ hybridisation (FISH) (data not shown). ERBB2-negative tumors revealed protein expression levels between 100 and 260 ng/mg.

### 3.4. Immunoassay of pYShcA and PSShcA Levels

Black 96–well microtiter plates (Nunc Black MaxiSorp Surface; Nalgen Nunc International, Rochester, NY) were coated with anti-Shc antibody, Cat. No. 06-203 (Upstate Biotechnology, Lake Placid, NY) at a concentration of 3 mg/mL of coating buffer in a volume of 100 µL/well and kept at 4 °C overnight. For determination of phosphorylated ShcA, tumor extracts were prepared as described as above in the presence of Na_3_VO_4_. Before sample applications, the coated microtiters were washed five times with 200 µL/well of washing and then blocked for 2 h at room temperature with 250 µL of blocking buffer. The blocked wells were washed five times with 300 µL of blocking buffer and then 50 µL of the diluted tumor membrane extracts or reference material was added to the wells and incubated overnight at 4 °C. As a reference for each assay, a cell extract of SKBr3 cells, stimulated as described above, was used. For use in ELISAs, the SKBr3 cell membrane extract was sequentially diluted with sample dilution buffer at ratios of 1×, 0.75×, 0.5×, 0.25×, 0.125×, and 0.025×, and then 100 µL aliquots were incubated on each microtiter plate together with the tumor tissue extracts and blanks (containing only dilution buffer). After incubation of the samples and references, the wells were washed five times with 300 µL washing buffer at room temperature to eliminate unbound particles. Biotinylated PhosphoSerine Antibody Q5, Cat. No. 37430 or alkaline phosphatase (AP) conjugated PhosphoTyrosine antibody PY20, Cat. No. 03-7722 (Zymed Laboratories Inc., South San Francisco, CA) was added to the wells and incubated for 2 h at room temperature. Complex was detected with AP-conjugated streptavidin, diluted in conjugate diluent for 1 h at room temperature. AP activity was detected with CDP Star substrate (Tropix, Bedford, MA) in a glow luminometer. The response data of the series of diluted reference material were fitted and the curve was used for quantification of the tumor extracts. The value of the undiluted SKBr3 extract was denominated 1 U/mL.

### 3.5. Statistical Methods

Correlations between continuous values were assessed by the Spearman rank correlation coefficient (*r*_s_). Statistical significance between pShcA and dichotomous variables was calculated using Mann-Whitney *U* test. In the present study patients were dichotomized and defined as pShcA-negative/positive according to median or third quartile value of pShcA. Relationships between categorical data were assessed using Fisher’s exact test. Survival curves were estimated by the Kaplan–Meier method and statistical significance compared by means of log-rank test, using S-Plus statistical software (Insightful, version 6); these data were also confirmed using STATA 10 statistical software.

## 4. Conclusions

Using a novel CLISA assay, we demonstrate that increased levels of phosphorylated ShcA assay correlates with good prognosis in selected primary breast cancer with lower ER expression levels. Our results indicate the need for further studies on the role of ShcA phosphorylation in breast cancer, using a larger patient collective with a longer follow-up time. It would be also important to develop assays recognizing particular phosphorylation sites on the ShcA protein or upstream factors in order to elucidate the role of specific sites. Moreover, the direct correlation between the ShcA phosphorylation and Erk1/2 phosphorylation should be further investigated. Of particular clinical relevance will be the analysis of the possible predictive value of pShcA toward antiestrogen, anti-ErbB2 as well as other targeted therapies.

## References

[1] Cicenas J., Urban P., Küng W., Vuaroqueaux V., Labuhn M., Wight E., Eppenberger U., Eppenberger-Castori S. (2005). Increased level of phosphorylated akt measured by chemiluminescence-linked immunosorbent assay is a predictor of poor prognosis in primary breast cancer overexpressing ErbB-2. Breast Cancer Res..

[2] Cicenas J., Urban P., Vuaroqueaux V., Labuhn M., Küng W., Wight E., Mayhew M., Eppenberger U., Eppenberger-Castori S. (2006). Phosphorylation of tyrosine 1248-ERBB2 measured by chemiluminescence-linked immunoassay is an independent predictor of poor prognosis in primary breast cancer patients. Eur. J. Cancer.

[3] Bonfini L., Migliaccio L., Pelicci G, Lanfrancone L., Pelicci P.G. (1996). Not all Shc’s roads lead to Ras. Trends Biochem. Sci..

[4] Marshall M.S. (1995). Ras target proteins in eukaryotic cells. FASEB.

[5] Salcini AE., McGlade J., Pelicci G., Nicoletti I., Pawson TP., Pelicci PG. (1994). Formation of Shc–Grb2 complexes is necessary to induce neoplastic transformation by overexpression of Shc proteins. Oncogene.

[6] Gotoh N., Toyoda M., Shibuya M. (1997). Tyrosine phosphorylation sites at amino acids 239 and 240 of Shc are involved in epidermal growth factor-induced mitogenic signalling that is distinct from Ras/mitogen-activated protein kinase activation. Mol. Cell Biol..

[7] Lanfrancone L., Pelicci G., Brizzi M.F., Aronica M.G., Casciari C., Giuli S., Pegoraro L., Pawson T., Pelicci P.G., Arouica M.G. (1995). Overexpression of Shc proteins potentiates the proliferative response to the granulocyte-macrophage colony-stimulating factor and recruitment of Grb2/SoS and Grb2/p140 complexes to the beta receptor subunit. Oncogene.

[8] Lai K.M., Pawson T. (2000). The ShcA phosphotyrosine docking protein sensitizes cardiovascular signalling in the mouse embryo. Genes Dev..

[9] Migliaccio E., Mele S., Salcini A.E., Pelicci G., Lai K.M., Superti-Furga G., Pawson T., Di Fiore P.P., Lanfrancone L., Pelicci P.G. (1997). Opposite effects of the p52shc/p46shc and p66shc splicing isoforms on the EGF receptor-MAP kinase-fos signalling pathway. EMBO J..

[10] Okada S., Kao A.W., Ceresa B.P., Blaikie P., Margolis B., Pessin J.E. (1997). The 66-kDa Shc isoform is a negative regulator of the epidermal growth factor-stimulated mitogen-activated protein kinase pathway. J. Biol. Chem..

[11] Le S., Connors T.J., Maroney A.C. (2001). c-Jun N-terminal kinase specifically phosphorylates p66ShcA at serine 36 in response to ultraviolet irradiation. J. Biol. Chem..

[12] Cicenas J. (2008). The potential role of Akt phosphorylation in human cancers. Int. J. Bio. Markers.

[13] Wu Y., Mohamed H., Chillarm R., Ali I., Clayton S., Slamon D., Vadgama J.V. (2008). Clinical significance of Akt and HER2/neu overexpression in African-American and Latina women with breast cancer. Breast Cancer Res..

[14] Cicenas J. (2007). The potential role of the EGFR/ERBB2 heterodimer in breast cancer. Expert. Opin. Ther. Pat..

[15] Xia W., Chen J.S., Zhou X., Sun P.R., Lee D.F., Liao Y., Zhou B.P., Hung M.C. (2004). Phosphorylation/cytoplasmic localization of p21Cip1/WAF1 is associated with HER2/neu overexpression and provides a novel combination predictor for poor prognosis in breast cancer patients. Clin. Cancer Res..

[16] Clarke R.B. (2003). p27KIP1 phosphorylation by PKB/Akt leads to poor breast cancer prognosis. Breast Cancer Res..

[17] Eppenberger-Castori S., Kueng W., Benz C., Caduff R., Varga Z., Bannwart F., Fink D., Dieterich H., Hohl M., Müller H. (2001). Prognostic and predictive significance of ErbB-2 breast tumor levels measured by enzyme immunoassay. J. Clin. Oncol..

[18] Milde-Langosch K., Bamberger A.M., Rieck G., Grund D., Hemminger G., Müller V., Löning T. (2005). Expression and prognostic relevance of activated extracellular-regulated kinases (ERK1/2) in breast cancer. Br. J. Cancer.

[19] Svensson S., Jirström K., Rydén L., Roos G., Emdin S., Ostrowski M.C., Landberg G. (2005). ERK phosphorylation is linked to VEGFR2 expression and Ets-2 phosphorylation in breast cancer and is associated with tamoxifen treatment resistance and small tumors with good prognosis. Oncogene.

[20] Bergqvist J., Elmberger G., Ohd J., Linderholm B., Bjohle J., Hellborg H., Nordgren H., Borg A.L., Skoog L., Bergh J. (2006). Activated ERK1/2 and phosphorylated oestrogen receptor alpha are associated with improved breast cancer survival in women treated with tamoxifen. Eur. J. Cancer.

[21] Schmitt M., Mengele K., Schueren E., Sweep F.C., Foekens J.A., Brünner N., Laabs J., Malik A., Harbeck N., European Organisation for Research and Treatment of Cancer Pathobiology Group (2007). European Organisation for Research and Treatment of Cancer (EORTC) Pathobiology Group standard operating procedure for the preparation of human tumour tissue extracts suited for the quantitative analysis of tissue-associated biomarkers. Eur. J. Cancer.

